# A comparison between children and adolescents with autism spectrum disorders and healthy controls in biomedical factors, trace elements, and microbiota biomarkers: a meta-analysis

**DOI:** 10.3389/fpsyt.2023.1318637

**Published:** 2024-01-09

**Authors:** Ping Lin, Qianwen Zhang, Junyu Sun, Qingtian Li, Dan Li, Mengyuan Zhu, Xiaomei Fu, Ling Zhao, Mengxia Wang, Xiaoyan Lou, Qing Chen, Kangyi Liang, Yuxin Zhu, Caiwei Qu, Zhenhua Li, Peijun Ma, Renyu Wang, Huafen Liu, Ke Dong, Xiaokui Guo, Xunjia Cheng, Yang Sun, Jing Sun

**Affiliations:** ^1^Department of Medical Microbiology and Parasitology, School of Basic Medical Sciences, Fudan University, Shanghai, China; ^2^Department of Clinical Laboratory, Shanghai Mental Health Center, Shanghai Jiao Tong University School of Medicine, Shanghai, China; ^3^Key Laboratory of Digital Technology in Medical Diagnostics of Zhejiang Province, Hangzhou, China; ^4^Hangzhou Calibra Diagnostics, Hangzhou, China; ^5^Department of Psychosis Studies, Institute of Psychiatry, Psychology and Neuroscience, King’s College London, London, United Kingdom; ^6^College of Health Science and Technology, Shanghai Jiao Tong University School of Medicine, Shanghai, China; ^7^Institute for Global Health, Shanghai Jiao Tong University School of Medicine, Shanghai, China; ^8^Institute of Arthritis Research, Shanghai Academy of Chinese Medical Sciences, Shanghai, China; ^9^School of Medicine and Dentistry, Institute for Integrated Intelligence and Systems, Griffith University, Gold Coast Campus, Gold Coast, QLD, Australia; ^10^Charles Sturt University, Orange, NSW, Australia

**Keywords:** autism spectrum disorder, biomarkers, biomedical, trace elements, microbiota

## Abstract

**Introduction:**

Autism spectrum disorder (ASD) is a multifaceted developmental condition that commonly appears during early childhood. The etiology of ASD remains multifactorial and not yet fully understood. The identification of biomarkers may provide insights into the underlying mechanisms and pathophysiology of the disorder. The present study aimed to explore the causes of ASD by investigating the key biomedical markers, trace elements, and microbiota factors between children with autism spectrum disorder (ASD) and control subjects.

**Methods:**

Medline, PubMed, ProQuest, EMBASE, Cochrane Library, PsycINFO, Web of Science, and EMBSCO databases have been searched for publications from 2012 to 2023 with no language restrictions using the population, intervention, control, and outcome (PICO) approach. Keywords including “autism spectrum disorder,” “oxytocin,” “GABA,” “Serotonin,” “CRP,” “IL-6,” “Fe,” “Zn,” “Cu,” and “gut microbiota” were used for the search. The Joanna Briggs Institute (JBI) critical appraisal checklist was used to assess the article quality, and a random model was used to assess the mean difference and standardized difference between ASD and the control group in all biomedical markers, trace elements, and microbiota factors.

**Results:**

From 76,217 records, 43 studies met the inclusion and exclusion criteria and were included in this meta-analysis. The pooled analyses showed that children with ASD had significantly lower levels of oxytocin (mean differences, MD = −45.691, 95% confidence interval, CI: −61.667, −29.717), iron (MD = −3.203, 95% CI: −4.891, −1.514), and zinc (MD = −6.707, 95% CI: −12.691, −0.722), lower relative abundance of *Bifidobacterium* (MD = −1.321, 95% CI: −2.403, −0.238) and *Parabacteroides* (MD = −0.081, 95% CI: −0.148, −0.013), higher levels of c-reactive protein, CRP (MD = 0.401, 95% CI: 0.036, 0.772), and GABA (MD = 0.115, 95% CI: 0.045, 0.186), and higher relative abundance of *Bacteroides* (MD = 1.386, 95% CI: 0.717, 2.055) and *Clostridium* (MD = 0.281, 95% CI: 0.035, 0.526) when compared with controls. The results of the overall analyses were stable after performing the sensitivity analyses. Additionally, no substantial publication bias was observed among the studies.

**Interpretation:**

Children with ASD have significantly higher levels of CRP and GABA, lower levels of oxytocin, iron, and zinc, lower relative abundance of *Bifidobacterium* and *Parabacteroides*, and higher relative abundance of *Faecalibacterium*, *Bacteroides,* and *Clostridium* when compared with controls. These results suggest that these indicators may be a potential biomarker panel for the diagnosis or determining therapeutic targets of ASD. Furthermore, large, sample-based, and randomized controlled trials are needed to confirm these results.

## Introduction

Autism spectrum disorder (ASD) is a multifaceted developmental condition that commonly appears during early childhood. It is marked by difficulties in social interaction, challenges in communication, and the presence of repetitive behaviors or fixations (1). According to the estimation by the American Centers for Disease Control and Prevention Center (CDC), one in 44 children aged 8 years was diagnosed with autism in 2018 (2). The prevalence of autism was approximately 1% globally (3). The risk in boys is 3–4 times higher than that in girls, and male autistic patients tend to exhibit more obvious signs than female counterparts (4). The current clinical diagnosis of autism is largely dependent on the fifth version of Diagnostic and Statistical Manual of Mental Disorder (DSM-5) by the American Psychiatric Association (1). ASD is usually first diagnosed in toddlerhood, with many of the most obvious signs (e.g., stop acquiring or losing previously gained skills) presenting around 2–3 years old; however, clear predictions of later cognitive impairment are sometimes difficult in children at 2–3 years of age (1).

Despite extensive research efforts, the etiology of ASD remains multifactorial and not yet fully understood (5–9). In recent years, studies in biomarkers have found that patients with ASD have a higher level of inflammatory factors, such as C-reactive protein (CRP) (10, 11), a lower tracer elements level including iron (Fe) (12, 13) and zinc (Zn) (14, 15), and a lower abundance of beneficial microbiota bacteria (16–19) than healthy patients. The identification of biomarkers may provide insights into the underlying mechanisms and pathophysiology of the disorder (20–22). However, there is no study that has systematically assessed the characteristics of ASD patients in comparison with healthy people. This study aimed to fill in this research gap by comprehensively evaluating a wide range of potential biomarkers associated with ASD, including biomedical markers, trace elements, and microbiota factors, using the meta-analysis method. This method included potential biomedical markers (oxytocin, γ-aminobutyric acid, i.e., GABA, serotonin, CRP, and IL-6), trace elements (Fe, Zn, and Cu), and microbiota factors (*Bifidobacterium*, *Faecalibacterium*, *Parabacteroides*, *Bacteroides*, and *Clostridium*) in ASD. The inclusion of specific biomarkers in this study was predicated upon their established roles within biological pathways pertinent to ASD. Oxytocin, GABA, serotonin, CRP, and IL-6 were selected due to their documented involvement in neurodevelopmental processes, neurotransmission modulation, and immune system regulation—all of which are fundamental elements intertwined with ASD pathophysiology (10, 23–25). These biomarkers are integral to understanding the neurobiological substrates governing social cognition, emotional regulation, and behavioral responses, all of which are significantly impaired in individuals with ASD. Furthermore, the incorporation of trace elements, i.e., Fe, Zn, and Cu, stems from their crucial roles as cofactors in essential enzymatic reactions pivotal for brain development and synaptic functioning (26–29). Perturbations in their levels have exhibited correlations with ASD-related traits, necessitating their examination within this context. Additionally, the exploration of microbiota factors, including *Bifidobacterium*, *Faecalibacterium*, *Parabacteroides*, *Bacteroides*, and *Clostridium*, aligns with burgeoning research elucidating the bidirectional communication between the gut microbiome and the central nervous system (30–34). These factors potentially influence neurodevelopment and behavior, prompting their investigation in relation to ASD. By examining and synthesizing the existing literature on these biomarkers, this study aimed to enhance the understanding of the biological underpinnings of ASD and potentially contribute to the development of improved diagnostic and therapeutic approaches.

## Methods

### Search strategy and eligibility criteria

This meta-analysis was prepared in accordance with the preferred reporting items for systematic reviews and meta-analyses (PRISMA) standards (35) (Supplementary eMethods 1). Medline, PubMed, ProQuest, EMBASE, Cochrane Library, PsycINFO, Web of Science, and EMBSCO databases have been searched for publications from 2012 to 2023 with the following keywords: “autism spectrum disorder,” “oxytocin,” “GABA,” “Serotonin,” “CRP,” “IL-6,” “Fe,” “Zn,” “Cu,” and “gut microbiota” (Supplementary eMethods 2).

Using population, intervention, control, and outcome (PICO) approach, the study included the following criteria: (i) P: studies conducted with ASD participants aged 18 years or younger, (ii) C: assessment of participants with ASD and controls, and (iii) O: reporting oxytocin, GABA, serotonin, CRP, IL-6, Fe, Zn, Cu, and gut microbiota levels with available data for the meta-analysis. Studies with full-text available were included regardless of language. As intervention studies are not available, the present study focused on observational studies, including cross-sectional, case-control, or prospective cohort studies. Any studies that did not meet the aforementioned criteria, e.g., P: patients older than 18 years old; C: control groups who are not healthy or have other mental disorders; and O: outcomes focusing on other types of biomarkers, were excluded.

### Data extraction and study quality assessment

The characteristics of the studies, including the following data, were extracted from each study: first author’s surname, year of publication, country, study design, total population, number of male participants, mean age, age range, biomarker type, contributed biomarker, study outcome, and statistics for meta-analysis (mean, standard deviation, and participant number in ASD and control groups).

Finally, for outcomes including biomedical markers and trace elements, the mean and standard deviation of concentrations were extracted and for microbiota factors, the genera level of relative abundance (%) was extracted.

The Joanna Briggs Institute (JBI) critical appraisal checklist was used to assess the article quality (36). The total JBI scores possible for a cohort study is 12 (37), for case-control study is 10 (38), and for cross-sectional study is 8 (39).

### Statistical analysis

For biomedical markers and trace elements, the mean and standard deviation of concentrations were collected; for microbiota factors, the genera level of relative abundance (%) was recorded. Mean differences (MDs) were used as the primary index of comparative results between ASD and the control group, and standard mean differences (SMDs) were used as the effect size. Heterogeneity across the studies was examined using the *I*^2^ statistics. Low heterogeneity was indicated by *I*^2^ less than 25%, moderate heterogeneity by *I*^2^ of approximately 50%, and substantial heterogeneity by *I*^2^ of 75% (40). The significance was quantified with Cochrane’s *Q* statistics. Random-effects models were chosen if heterogeneity by *I*^2^ is approximately 50% across studies resulting from differences in subjects and measurements. Sensitivity analysis was performed to avoid any single study influencing the results of the meta-analysis. To access the publication bias, Egger’s test was performed (41), and forest plots were performed to visualize the results. A two-tailed *p*-value of <0.05 was considered significant. The analyses were performed in a comprehensive meta-analysis (version 3) (42).

## Results

### Study selection

**Figure 1:**
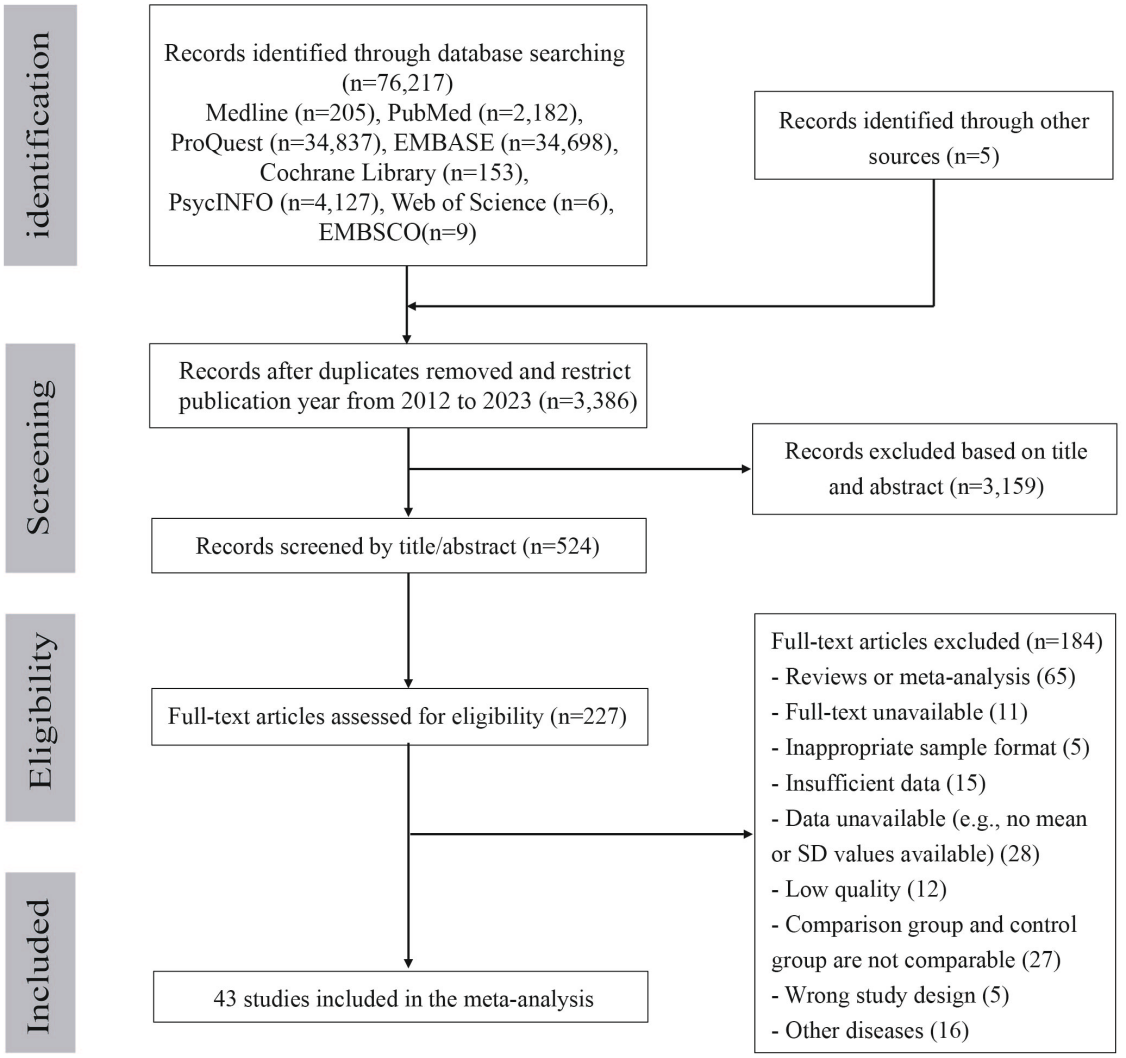
Flow diagram of study selection.

The selection process for the included studies is shown in Figure 1. From the 76,217 potentially eligible articles published between January 1967 and May 2023, 3,386 publications remained after removing duplicates and restricting the publication year from 2012 to 2023. Following the screening of the title and abstracts, the full-text of 227 publications were assessed, and 184 publications were excluded for the following reasons: review or meta-analysis, unavailability of the full-text, inappropriate sample format, wrong study design, wrong study population, insufficient data, low quality, incomparability of the comparison and control groups, and other diseases. Finally, 43 publications satisfied the eligibility criteria and were included in the meta-analysis.

### Study characteristics

The study characteristics are summarized in Table 1. A total of 17 articles had the results of biomedical markers (10, 11, 24, 43–56); 8 articles had the results of trace elements (12–15, 57–60); 16 articles had the results of microbiota factors (16–19, 63–74); and 2 articles had the results for both biomedical markers and microbiota factors (61, 62). These studies were performed in 16 countries, including 16 studies in China (10, 11, 14, 17, 18, 47, 55, 58, 61, 63, 64, 66, 67, 69, 70), 5 studies in the USA (52, 59, 71, 72, 74), 4 studies in Iraq (43, 44, 48, 49), 3 studies in Egypt (13, 45, 51), 3 studies in Italy (16, 19, 68), 2 studies in Jordan (15, 53), 2 studies in Turkey (54, 56), and 1 study each in Ecuador (62), India (73), Japan (46), Malaysia (60), Qatar (12), Russia (57), Saudi Arabia (24), Spain (65), and Tunisia (50). In this pooled review, 35 articles were case-control studies and their JBI scores ranged from 6 to 10; 7 articles were cross-sectional studies and their JBI scores ranged from 5 to 6; and 1 article was a cohort study and its JBI score was 10.

**Table 1:** Study characteristics of included studies.

Study	Country	Study design	Total population	Number of boys	Boys %	Mean age	Age range	Biomarkers type	Contributed biomarkers	Study outcomes	JBI scores	Total JBI scores possible
Abdulamir 2016 (43)	Iraq	Case-control	86	86	100%	7.28	3 to 13	Biomedical markers	Oxytocin	Oxytocin: ASD↓	8	10
Abdulamir 2018 (44)	Iraq	Cross-sectional	86	86	100%	7.28	3 to 13	Biomedical markers	Serotonin	Serotonin: ASD↑	6	8
Alabdali 2014 (24)	Saudi Arabia	Cross-sectional	82	82	100%	7.20	3 to 12	Biomedical markers	GABA, serotonin, oxytocin	GABA: ASD↑; serotonin, oxytocin: ASD↓	6	8
El-Ansary 2019 (45)	Egypt	Case-control	40	22	55%	3.34	3 to 4	Biomedical markers	GABA	GABA: ASD↓	8	10
Tanaka 2020 (46)	Japan	Case-control	20	15	75%	9.6	8 to 13	Biomedical markers	Oxytocin	Oxytocin: ASD↑	8	10
Huang 2021 (47)	China	Case-control	83	63	76%	4.9	3 to 7	Biomedical markers	Oxytocin	Oxytocin: ASD↓	8	10
Al-Ali 2022 (48)	Iraq	Case-control	120	N/A	N/A	7.08	3 to 15	Biomedical markers	Oxytocin	Oxytocin: ASD↑	6	10
Mossa 2020 (49)	Iraq	Case-control	80	N/A	N/A	N/A	3 to 13	Biomedical markers	Oxytocin	Oxytocin: ASD↓	6	10
Chamtouri 2023 (50)	Tunisia	Case-control	56	44	78.6	N/A	4 to 10	Biomedical markers	GABA	GABA: ASD↑	6	10
Mostafa 2021 (51)	Egypt	Case-control	44	11	25%	5.75	3 to 11	Biomedical markers	Serotonin	Serotonin: ASD↑	9	10
Zuniga-Kennedy 2022 (52)	USA	Case-control	17	N/A	N/A	10.5	4 to 18	Biomedical markers	Serotonin	Serotonin: ASD↑	7	10
Alzghoul 2019 (53)	Jordan	Cross-sectional	166	108	65%	6.28	<12	Biomedical markers	IL-6	IL-6: ASD↑	5	8
Esnafoglu 2022 (54)	Turkey	Cross-sectional	252	192	76%	7.34	N/A	Biomedical markers	CRP	CRP: ASD↑	6	8
Ning 2019 (10)	China	Case-control	204	160	78%	4.5	N/A	Biomedical markers	CRP, IL-6	CRP, IL-6: ASD↑	10	10
Shen 2021 (55)	China	Case-control	83	62	75%	4.3	3 to 8	Biomedical markers	IL-6	IL-6: ASD↑	9	10
Zhao 2015 (11)	China	Case-control	160	128	80%	3.69	N/A	Biomedical markers	Hs-CRP	CRP: ASD↑	10	10
Kartalcı 2022 (56)	Turkey	Cohort	70	52	74%	8	3 to 12	Biomedical markers	IL-6	IL-6: ASD↑	10	12
Bener 2017 (12)	Qatar	Case-control	616	N/A	N/A	5.51	<8	Trace element	Fe	Fe: ASD↓	7	10
Li 2014 (14)	China	Case-control	120	96	80%	3.78	N/A	Trace element	Zn, Cu	Zn: ASD↓; Cu: ASD↑	8	10
Skalny 2016 (57)	Russia	Case-control	96	48	50%	6.55	N/A	Trace element	Fe, Cu, Zn	Fe: ASD↓; Zn: ASD↑	7	10
Wu 2018 (58)	China	Case-control	254	194	76%	4.95	2 to 10	Trace element	Fe, Cu, Zn	Fe: ASD↑; Cu, Zn: ASD↓	8	10
Mehta 2021 (59)	USA	Case-control	129	89	69%	3	2 to 4	Trace element	Zn	Zn: ASD↓	7	10
Higazi 2021 (13)	Egypt	Case-control	85	54	64%	8.35	4 to 13	Trace element	Fe	Fe: ASD↓	6	10
Abd Wahil 2022 (60)	Malaysia	Case-control	155	41	26%	3	2 to 6	Trace element	Fe	Fe: ASD↓	8	10
Rashaid 2021 (15)	Arab	Case-control	107	87	81%	7.46	2 to 12	Trace element	Fe, Cu, Zu	Fe, Cu, Zn: ASD↓	7	10
Wang 2020 (61)	China	Case-control	50	46	92%	4.4	2 to 8	Biomedical markers and Microbiota markers	Serotonin, GABA, *Clostridium*	Serotonin: ASD↑; GABA: ASD↓; *Clostridium*: ASD↑	8	10
Zurita 2020 (62)	Ecuador	Case-control	60	56	93%	8.65	N/A	Biomedical markers, microbiota markers	IL-6, *Bacteroides*	*Bacteroides*: ASD↑	8	10
Ding 2020 (63)	China	Case-control	127	98	77%	3.33	N/A	Microbiota markers	*Bacteroides*, *Faecalibacterium*	*Bacteroides*, *Faecalibacterium*: ASD↓	8	10
Xie 2022 (64)	China	Case-control	204	166	81%	4.33	N/A	Microbiota markers	*Bacteroides*, *Faecalibacterium*	*Bacteroides*: ASD↓; *Faecalibacterium*: ASD↑	8	10
Ding 2021 (17)	China	Case-control	45	33	73%	5.55	N/A	Microbiota markers	*Bacteroides*, *Faecalibacterium*	*Bacteroides*: ASD↓; *Faecalibacterium*: ASD↑	8	10
Plaza-Díaz 2019 (65)	Spain	Cross-sectional	115	N/A	N/A	N/A	2 to 6	Microbiota markers	*Faecalibacterium*, *Bacteroides*, *Clostridium*	*Clostridium*: ASD↑; *Faecalibacterium*, *Bacteroides*: ASD↓	6	8
Ma 2019 (66)	China	Cross-sectional	90	78	87%	7.15	6 to 9	Microbiota markers	*Bacteroides*, *Clostridium*, *Faecalibacterium*, *Parabacteroides*	*Bacteroides*, *Faecalibacterium*, *Clostridium*: ASD↑; *Parabacteroides*: ASD↓	6	8
Coretti 2018 (16)	Italy	Cross-sectional	25	17	68%	2.9	2 to 4	Microbiota markers	*Bacteroides*, *Faecalibacterium*, *Parabacteroides*	*Bacteroides*, *Faecalibacterium*, *Parabacteroides*: ASD↑	6	8
Strati 2017 (19)	Italy	Case-control	80	59	74%	8.5	3.6 to 17	Microbiota markers	*Bacteroides*, *Faecalibacterium*, *Clostridium*, *Parabacteroides*	*Faecalibacterium*, *Clostridium*: ASD↑; *Bacteroides*, *Parabacteroides:* ASD↓	8	10
Liu 2022 (67)	China	Case-control	50	36	72%	3.80	2 to 6.8	Microbiota markers	*Bacteroides*	*Bacteroides*: ASD↓	8	10
Chiappori 2022 (68)	Italy	Case-control	12	8	67%	13	6 to 20	Microbiota markers	*Bacteroides*	*Bacteroides*: ASD↑	8	10
Zou 2020 (69)	China	Case-control	60	38	63%	4.5	2 to 6.5	Microbiota markers	*Bacteroides*, *Clostridium*	*Bacteroides*: ASD↑; *Clostridium*: ASD↓	6	10
Dan 2020 (18)	China	Case-control	286	257	89%	4.9	2 to 13	Microbiota markers	*Bacteroides*, *Parabacteroides*, *Clostridium*	*Bacteroides*, *Parabacteroides*, *Clostridium:* ASD↓	7	10
Sun 2019 (70)	China	Case-control	15	12	80%	N/A	3 to 12	Microbiota markers	*Bacteroides*	*Bacteroides:* ASD↓	8	10
Maigoro 2021 (71)	USA	Case-control	57	N/A	N/A	N/A	N/A	Microbiota markers	*Bacteroides*	*Bacteroides*: ASD↑	6	10
Kang 2017 (72)	USA	Case-control	38	34	89%	10.8	7 to 16	Microbiota markers	*Bacteroides*, *Clostridium*	*Bacteroides*, *Clostridium*: ASD↑	8	10
Pulikkan 2018 (73)	India	Case-control	54	43	80%	9.5	3 to 16	Microbiota markers	*Bacteroides*	*Bacteroides*: ASD↓	9	10
Kang 2013 (74)	USA	Case-control	40	35	88%	6.7	3 to 16	Microbiota markers	*Bacteroides*, *Clostridium*	*Bacteroides*, *Clostridium*: ASD↑	10	10

GABA, gamma-aminobutyric acid; IL-6, interleukin-6; CRP, C-reactive protein; Cu, cooper; Fe, iron; Zn, zinc; N/A, not available; JBI, Joanna Briggs Institute; ↑, indicating the concentration of the biomedical marker or the trace element, or the genera level of relative abundance is higher in the ASD than in the controls; ↓, indicating the concentration of the biomedical marker or the trace element, or the genera level of relative abundance is lower in the ASD than in the controls.

### Meta-analysis results

#### Biomedical markers

**Figure 2:**
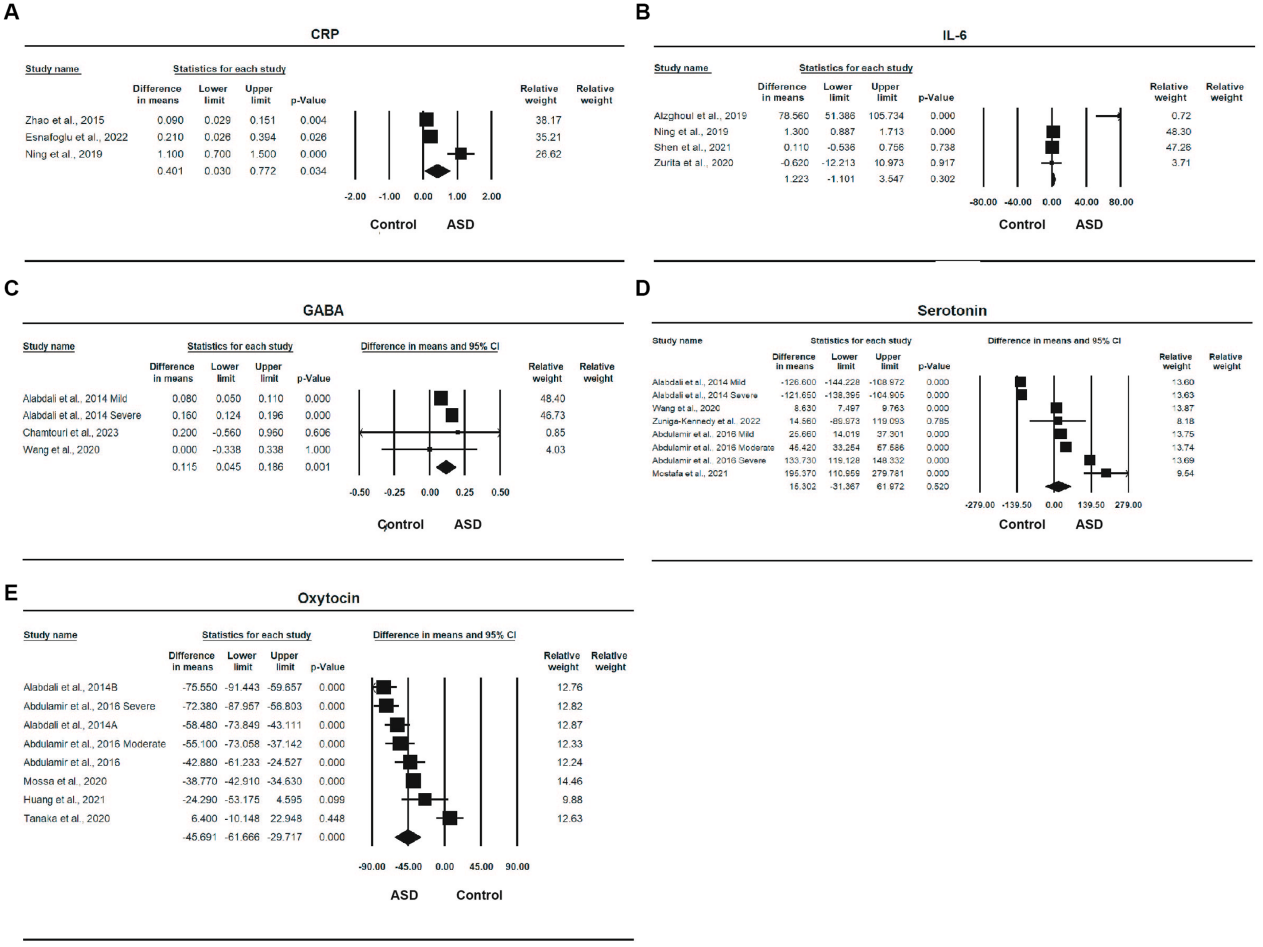
Forest plots of biomedical markers between ASD and control; **(A)** CRP; **(B)** IL-6; **(C)** GABA; **(D)** Serotonin; **(E)** Oxytocin.

Three studies (10, 11, 54), with a total of 616 participants, provided sufficient information for a meta-analysis of CRP (Table 2). There was a statistically significant difference in the mean CRP concentration between ASD and control, with an overall mean difference of 0.401 (95% CI: 0.036, 0.772, *p* = 0.034), indicating that the ASD group had a higher CRP concentration than the healthy control group. The effect size was moderate, and the SMD was 0.497 (95% CI: 0.219, 0.775, *p* < 0.001). The heterogeneity was significant (*I*^2^ = 91.968%, *p* < 0.0001). The forest plot of the effect is shown in Figure 2A.

**Table 2:** Meta-analysis of immune and gut microbiota characteristics.

Characteristics	Studies (*n*)	Participants (*N*)	Mean differences		Standardized mean differences		Heterogeneity		
			Mean differences (95% CI)	*p*-value	Standardized mean differences (95% CI)	*p*-value	*Q* test (df)	*p*-value	*I*^2^ (%)
CRP	3	616	0.401 (0.036, 0.772)^*^	0.034	0.497 (0.219, 0.775)^***^	<0.0001	24.901 (2)^***^	<0.0001	91.968
IL-6	4	510	1.223 (−1.101, 3.547)	0.302	0.485 (0.034, 0.936)^*^	0.035	40.662 (3)^***^	<0.0001	92.622
GABA	3	166	0.115 (0.045, 0.186)^**^	0.001	1.163 (−0.022, 2.347)	0.054	11.656 (3)^***^	0.009	74.26
Serotonin	5	283	15.302 (−31.367, 61.972)	0.52	0.825 (−1.127. 2.776)	0.407	802.682 (7)^***^	<0.0001	99.128
Oxytocin	5	419	−45.691 (−61.667, −29.717)^***^	<0.0001	−1.849 (2.796, −0.903)^***^	<0.0001	74.833 (7)^***^	<0.0001	90.646
Cu	4	577	0.293 (−1.349, 1.935)	0.726	0.059 (−0.368, 0.485)	0.788	19.429 (3)^***^	<0.0001	84.559
Fe	6	1,313	−3.203 (−4.891, −1.514)^***^	<0.0001	−0.375 (−0.743, −0.007)^*^	0.046	100.645 (5)^***^	<0.0001	95.032
Zn	5	706	−6.707 (−12.691, −0.722)^*^	0.028	−0.498 (−0.958, −0.037)^*^	0.034	80.898 (4)^***^	<0.0001	95.055
*Bifidobacterium*	11	1,067	−1.321 (−2.403, −0.238)^*^	0.017	−0.311 (−1.005, 0.383)	0.38	1225.316 (10)^***^	<0.0001	99.184
*Parabacteroides*	4	481	−0.081 (−0.148, −0.013)^*^	0.019	0.287 (−0.837, 1.410)	0.617	218.219 (3)^***^	<0.0001	98.625
*Faecalibacterium*	6	501	0.814 (−0.065, 1.693)	0.069	1.144 (0.296, 1.992)^**^	0.008	50.255 (5)^***^	<0.0001	90.051
*Bacteroides*	16	1,257	1.386 (0.717, 2.055)^***^	<0.0001	0.834 (0.197, 1.470)^**^	0.01	1393.282 (15)^***^	<0.0001	98.923
*Clostridium*	9	912	0.281 (0.035, 0.526)^*^	0.025	2.994 (1.724, 4.265)^***^	<0.0001	13777.581 (8)^***^	<0.0001	99.942

^*^p < 0.05; ^**^p < 0.01; ^***^p < 0.001. GABA, gamma-aminobutyric acid; IL-6, interleukin-6; CRP, C-reactive protein; Cu, cooper; Fe, iron; Zn, zinc; CI, confidence interval.

Four studies (10, 53, 55, 62), with a total of 510 participants, provided sufficient information for a meta-analysis of IL-6. The MD between ASD and control was 1.223 (95% CI, −1.101, 3.547, *p* = 0.302), and the ASD group had a higher level of IL-6 than the control group. The effect size was moderate, with an SMD of 0.485 (95% CI, 0.034, 0.936, *p* < 0.05). The heterogeneity was significant (*I*^2^ = 92.622%, *p* < 0.0001). The forest plot of the MD is shown in Figure 2B.

Three studies (24, 50, 61) involving a total of 166 participants provided sufficient information for a meta-analysis of GABA. There was a statistically significant difference in the mean GABA concentration between ASD and control, with an overall MD of 0.115 (95% CI: 0.045, 0.186, *p* = 0.001), and the ASD group had a higher GABA concentration than the control group. There effect size was large, the SMD was 1.163 (95% CI, −0.022, 2.347, *p* = 0.054), and the heterogeneity was also statistically significant (*I*^2^ = 74.260%, *p* < 0.0001). One study was removed due to sensitivity analysis (45). The forest plot of the effect is shown in Figure 2C.

Five studies (24, 44, 51, 52, 61) involving a total of 283 participants provided sufficient information for a meta-analysis of serotonin (Table 2). The MD between ASD and control was 15.302 (95% CI: −31.367, 61.972), indicating that the ASD group had a higher serotonin level than the control group, but the difference was not statistically significant (*p* = 0.520). The effect size was large, and the SMD was 0.825 (95% CI: −1.127, 2.776, *p* = 0.407); although the difference was also not statistically significant, heterogeneity was significant (*I*^2^ = 99.128%, *p* < 0.0001). The forest plot of serotonin is shown in Figure 2D.

Five studies (24, 43, 46, 47, 49) involving a total of 419 participants provided sufficient information for a meta-analysis of oxytocin. There was a statistically significant difference in the mean oxytocin concentration between ASD and control, with a mean difference of −45.691 (95% CI: −61.667, −29.717, *p* < 0.0001), and the ASD group had a lower oxytocin level than the control group and a very large effect size, with an SMD of −1.849 (95% CI: 2.796, −0.903, *p* < 0.0001). One study was removed due to sensitivity analysis (48). The heterogeneity was also significant (*I*^2^ = 90.646%, *p* < 0.0001). The forest plot of the effect is shown in Figure 2E.

#### Trace elements

**Figure 3:**
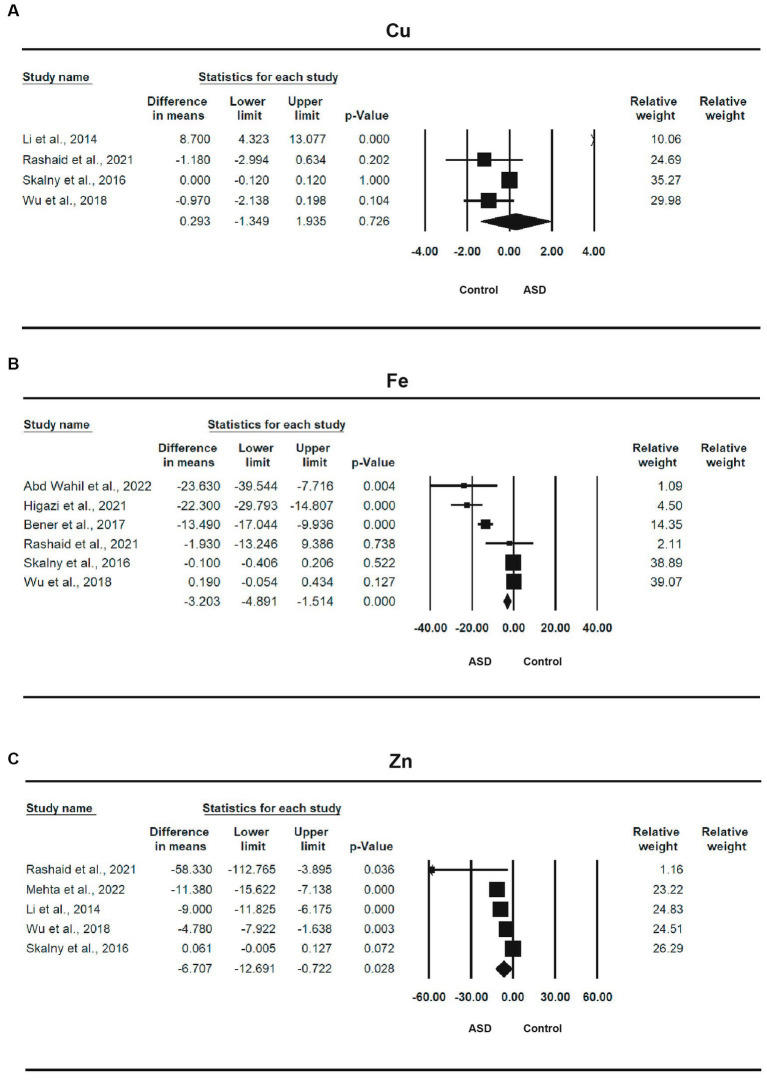
Forest plots of trace elements between ASD and control; **(A)** Cu; **(B)** Fe; **(C)** Zn.

Four studies (14, 15, 57, 58) involving a total of 577 participants provided sufficient information for a meta-analysis of Cu (Table 2). The MD between ASD and control was 0.293 (95% CI: −1.349, 1.935, *p* = 0.726), indicating that the ASD group had a high Cu level than the control and a small effect size, with an SMD of 0.059 (95% CI: −0.368, 0.485, *p* = 0.788). The heterogeneity was significant (*I*^2^ = 84.559%, *p* < 0.0001). The forest plot of the effect is shown in Figure 3A.

Six studies (12, 13, 15, 57, 58, 60) involving a total of 1,313 participants provided sufficient information for a meta-analysis of Fe (Table 2). There was a statistically significant difference in the mean iron concentration between ASD and control, with an MD of −3.203 (95% CI: −4.891, −1.514, *p* < 0.0001), and the ASD group had a lower iron level than the control and a smaller effect size, with a standardized mean difference of −0.375 (95% CI: −0.743, −0.007, *p* = 0.046). The heterogeneity was also significant (*I*^2^ = 95.032%, *p* < 0.0001). The forest plot of the effect is shown in Figure 3B.

Five studies (14, 15, 57–59), with a total of 706 participants, provided sufficient information for a meta-analysis of Zn (Table 2). There was a statistically significant difference in the mean zinc concentration between ASD and control, with a mean difference of −6.707 (95% CI: −12.691, −0.722, *p* = 0.028), and the ASD group had a lower zinc level than the control and a moderate effect size, with a standardized mean difference of −0.498 (95% CI: −0.958, −0.037, *p* = 0.034). The heterogeneity was also significant (*I*^2^ = 95.055%, *p* < 0.0001). The forest plot of the effect is shown in Figure 3C.

#### Microbiota factors

**Figure 4:**
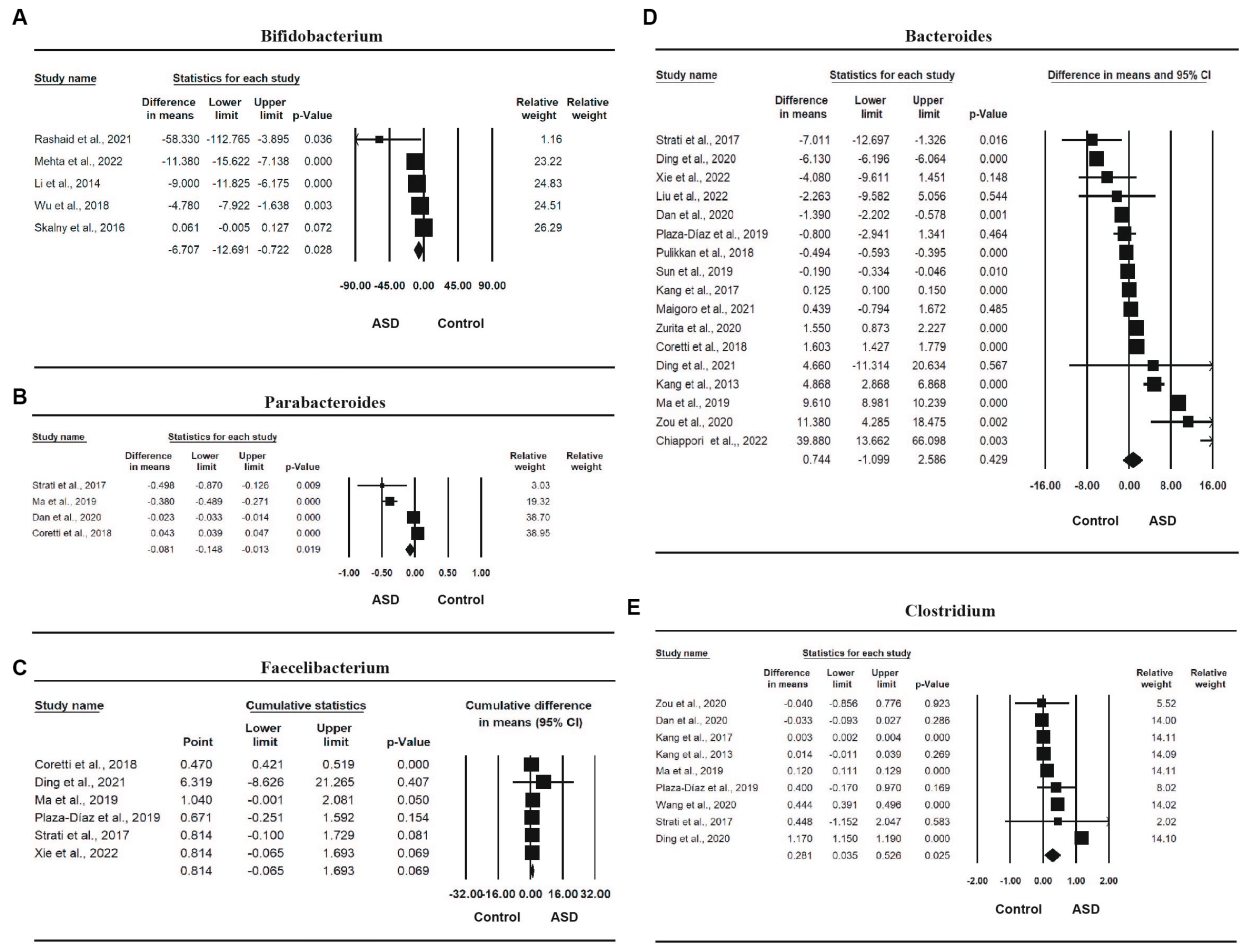
Forest plots of the relative abundance of microbiota factors between ASD and control; **(A)** *Bifidobacterium*; **(B)** *Faecalibacterium*; **(C)** *Parabacteroides*; **(D)** *Bacteroides*; **(E)** *Clostridium*.

Eleven studies (16–19, 61, 63–66, 72, 74) involving a total of 1,067 participants provided sufficient information for a meta-analysis of *Bifidobacterium* (Table 2). There was a statistically significant difference in the relative abundance of *Bifidobacterium* between ASD and control, with a mean difference of −1.321 (95% CI: −2.403, −0.238, *p* = 0.019), and the ASD group had a smaller abundance than the control group. The effect size was small, with an SMD of −0.311 [95% CI: −1.005, 0.383, which was not statistically significant (*p* = 0.380)]. The heterogeneity was also significant (*I*^2^ = 99.184%, *p* < 0.0001). The forest plot of the effect is shown in Figure 4A.

Four studies (16, 18, 19, 66) involving a total of 481 participants were included in the meta-analysis of *Parabacteroides*. There was a statistically significant difference in the relative abundance of *Parabacteroides* between ASD and control, with a mean difference of −0.081 (95% CI: −0.148, −0.013, *p* = 0.019), and the ASD group had a smaller abundance than the control group in *Parabacteroides*. The effect size was small, with an SMD of 0.287 (95% CI: −0.837, 1.410), which was not statistically significant (*p* = 0.617). One study was removed due to sensitivity analysis (65). The heterogeneity was also significant (*I*^2^ = 98.625%, *p* < 0.0001). The forest plot of the effect is presented in Figure 4B.

Six studies (16, 17, 19, 64–66) involving a total of 501 participants were included in the meta-analysis of *Faecalibacterium*. The mean difference of relative abundance between ASD and control was 0.814 (95% CI: −0.065, 1.693, *p* = 0.069), the ASD group had a higher abundance than the control group, and the effect size was large, with an SMD of 1.144 (95% CI: 0.296, 1.992), which was statistically significant (*p* = 0.008). One study (63) was removed due to sensitivity analysis, and another study (18) was removed due to publication bias and Egger’s test being less than 0.05. The heterogeneity was significant (*I*^2^ = 90.051%, *p* < 0.0001). The forest plot of the effect is shown in Figure 4C.

Sixteen studies (16–19, 62, 64–74) involving a total of 1,257 participants were included in the meta-analysis of *Bacteroides.* There was a statistically significant difference in the relative abundance of *Bacteroides* between ASD and control, with a mean difference of 1.386 (95% CI: 0.717, 2.055, *p* < 0.0001), and the ASD group had a higher abundance than the control group, and the effect size is large, with a standardized mean difference of 0.287 (95% CI: −0.837, 1.410, *p* = 0.01). One study was removed due to sensitivity analysis (63). The heterogeneity was also significant (*I*^2^ = 98.923%, *p* < 0.0001). The forest plot of the effect is shown in Figure 4D.

Finally, nine studies (18, 19, 61, 63, 65, 66, 69, 72, 74) involving a total of 912 participants were included in the meta-analysis of *Clostridium* (Table 2). There was a statistically significant difference in the relative abundance of *Clostridium* between ASD and control, with a mean difference of 0.281 (95% CI: 0.035, 0.526, *p* = 0.025), and the ASD group had a higher abundance than the control group, and a large effect size, with a standardized mean difference of 2.994 (95% CI: 1.724, 4.265, *p* < 0.0001). The heterogeneity was also significant (*I*^2^ = 99.942%, *p* < 0.0001). The forest plot of the effect is shown in Figure 4E.

#### Publication bias

Findings from the Egger’s test (Table 3), it can be suggested that there was no publication bias for all markers (CRP: *p* = 0.70; GABA: *p* = 1.00; serotonin: *p* = 0.99; oxytocin: *p* = 0.14; Cu: *p* = 0.56, Fe: *p* = 0.86; Zn: *p* = 0.92; *Bifidobacterium*: *p =* 0.71; *Parabacteroides*: *p* = 0.25; *Faecalibacterium*: *p =* 0.16; *Bacteroides*: *p =* 0.31; and *Clostridium*: *p =* 0.23), except IL-6 (*p =* 0.05).

**Table 3:** Results of publication bias.

Characteristics	Publication bias		
	Egger’s *t*	95% CI	*p*-value
CRP	0.509	(−176.440, 191.157)	0.7
IL-6	4.441^*^	(−17.069, −0.272)	0.047
GABA	0.006	(−7.867, 7.889)	0.996
Serotonin	0.018	(−11.605, 11.433)	0.985
Oxytocin	1.683	(−22.356, 4.134)	0.143
Cu	0.685	(−24.237, 33.417)	0.564
Fe	0.194	(−9.604, 11.047)	0.856
Zn	0.11	(−26.003, 24.258)	0.919
*Bifidobacterium*	0.381	(−9.747, 6.936)	0.712
*Parabacteroides*	1.601	(−27.894, 12.764)	0.251
*Faecalibacterium*	1.731	(−3.849, 16.605)	0.158
*Bacteroides*	1.044	(−2.897, 8.393)	0.314
*Clostridium*	1.318	(−15.396, 54.189)	0.229

^*^p < 0.05. GABA, gamma-aminobutyric acid; IL-6, interleukin-6; CRP, C-reactive protein; Cu, cooper; Fe, iron; Zn, zinc; CI, confidence interval.

## Discussion

The pooled effects based on our meta-analyses found that ASD youth have significantly higher levels of CRP and GABA, lower levels of oxytocin, iron, and zinc, lower relative abundance of *Bifidobacterium* and *Parabacteroides*, and higher relative abundance of *Faecalibacterium*, *Bacteroides*, and *Clostridium* when compared with control youth. To our knowledge, this is the first meta-analysis providing a wide range of potential biomarkers for children with ASD.

Inflammatory processes have gained increasing attention as potential contributors to the pathophysiology of ASD. Inflammation can impact brain development and function, and alterations in immune system markers have been observed in individuals with ASD (75, 76). CRP and IL-6 are two of the most commonly studied inflammatory markers and have shown associations with ASD in previous research (25, 77). CRP is an acute-phase protein that reflects systemic inflammation (78, 79). IL-6, a pro-inflammatory cytokine, is involved in immune reactions and synaptic plasticity (80, 81). In this meta-analysis, we have found that the CRP levels were significantly higher in ASD compared with controls, which is consistent with previous studies (77, 82). High levels of CRP could increase the blood-brain barrier (BBB) paracellular permeability, activating the microglia to impair the central nervous system (CNS) (83). Previous studies have pointed out that microglia play key roles in the pathogenesis of ASD (84–86). Thus, there might be a causal relationship between CRP levels and ASD, and CRP could be a potential biomarker for autism diagnosis. Elevated IL-6 levels have been documented not solely in autoimmune disorders but also in certain neurodegenerative conditions, such as Alzheimer’s disease, as well as diverse mental disorders (42, 87). Additionally, studies also reported that children who were later diagnosed with ASD have elevated levels of IL-6 during gestation, as IL-6 may have transferred across the placental barriers and accumulated in the fetuses (88, 89).

Neurotransmitters have also been implicated in the regulation of various neurodevelopmental processes, including social cognition, emotional regulation, and sensory integration (90, 91). Studies have suggested alterations in the levels and functioning of these neurotransmitters in individuals with ASD (92). Oxytocin, known for its role in social bonding and affiliation, has shown promise as a potential biomarker for ASD (93–95). Similarly, dysregulation of gamma-aminobutyric acid (GABA), the primary inhibitory neurotransmitter in the brain, has been implicated in the pathogenesis of ASD (96–98). Serotonin, involved in mood regulation and sensory processing, has also been the subject of investigation, with studies reporting variations in serotonin levels in individuals with ASD (52, 99, 100). In this meta-analysis, the ASD groups have significantly higher levels of GABA and lower levels of oxytocin compared with control groups. A preliminary meta-analysis has provided support for a potential link between autism spectrum disorder and variations in the oxytocin receptor gene, OXTR (94). However, this association does not reach the level of significance when considering the entire genome. Additionally, numerous studies have been conducted to explore the potential of oxytocin therapy in individuals with autism spectrum disorders (93, 101–105). Despite the fact that the majority of these investigations have yielded inconclusive results, oxytocin could be developed as a potential biomarker for autism diagnosis.

Trace elements play crucial roles in various biological processes, including neurotransmitter synthesis (106), antioxidant defense (107), and immune function (108). Imbalances in these trace elements have been reported in individuals with ASD, suggesting a potential link between their dysregulation and the pathogenesis of the disorder (109, 110). Iron deficiency, for instance, has been associated with impaired cognitive function and socioemotional difficulties observed in ASD (111, 112). Similarly, alterations in Zn and Cu levels have been reported, potentially affecting neurotransmitter systems and oxidative stress (113, 114). In this meta-analysis, we have found lower levels of Fe and Zn in the ASD groups. While it is acknowledged that there is a mineral imbalance in ASD, we anticipate the implementation of pertinent examinations and the establishment of standard levels for trace elements as potential biomarkers that can be valuable for diagnosing, preventing, and treating ASD.

Emerging research has also highlighted the potential involvement of the gut microbiome in ASD (115, 116). The gut microbiota, a diverse community of microorganisms residing in the gastrointestinal tract, is known to influence brain development and function through bidirectional communication with the central nervous system (117, 118). Altered microbial compositions and imbalances in microbial metabolites have been reported in individuals with ASD, suggesting a potential role for the gut microbiome as a biomarker and therapeutic target for the disorder (119–121). Specific bacterial genera, such as *Bifidobacterium*, *Faecalibacterium*, *Parabacteroides*, *Bacteroides*, and *Clostridium*, have been investigated in this meta-analysis. As a result, *Bifidobacterium* and *Parabacteroides* as protective bacteria showed a significantly lower abundance in the ASD group, while *Faecalibacterium*, *Bacteroides,* and *Clostridium* showed a significantly higher abundance in the ASD group, suggesting that there is indeed dysbiosis in ASD patients. Several studies have reported a lesser abundance of beneficial bacteria, such as *Bifidobacterium* in autism (16, 61, 65), leading to clinical trials with probiotic treatment (122–125). With the intervention of probiotics, children with ASD have shown improved autistic behavioral scores as well as gastrointestinal symptoms. In addition to the low abundance of beneficial bacteria, an increased abundance of bacteria such as *Faecalibacterium, Bacteroides*, and *Clostridium* in autism was also reported (126). Butyrate-producing bacteria, *Faecalibacterium,* were reported to affect physiological functions and homeostasis in the gut and were closely associated with autism core symptoms (127). *Bacteroides* are the main producers of propionic acid (PPA), which is one of the neurotoxic short-chain fatty acids, and have been reported to cause autistic characteristics in animal models (128). Additional studies suggested that *Bacteroides* are the distinguishing differences between autism and control children (129). Notwithstanding their non-invasive nature, *Clostridium* possess the ability to elicit deleterious effects on distant organs or tissues, such as the brain, by means of their secreted molecules traversing the intestinal barrier and spreading via the systemic circulation to remote locations, where they exert their biological activity (126). It has been hypothesized that there is a potential association between *Clostridium* and symptoms related to ASD (130), with a particular emphasis on the significance of *Clostridium*’s ability to form spores in relation to the recurrence of ASD symptoms (131).

This study has several limitations. First, this meta-analysis was based on observational studies; therefore, we cannot establish a causal relationship between the proposed markers and ASD. These changes in the levels of the abovementioned markers could be either a cause or a consequence of the pathogenesis of ASD. Second, some of the markers have limited numbers of studies, i.e., CRP only had three studies. While the results of the meta-analysis markers were consistent with previous studies (77, 82), further research is recommended. Third, heterogeneity was high in this meta-analysis. Due to the paucity of information from each of the included studies, we were unable to investigate the underlying confounding factors for the reason of high heterogeneity by doing subgroup analysis. For example, this meta-analysis encountered complexities in categorizing participants into children and adolescents due to heterogeneous age reporting across the included studies. Ambiguities in age ranges and missing mean age data hindered robust subgroup analyses, highlighting the need for standardized and comprehensive age reporting in future ASD biomarker studies to elucidate age-specific trends more effectively. Future studies will be conducted to systematically assess the randomized controlled trial on these markers, and subgroup analysis will be conducted to identify the sources of high heterogeneity.

Despite the limitations, this meta-analysis also has several major strengths. First, we conducted a comprehensive literature search through a list of electronic databases. Second, our literature selection workflow was based on rigorous inclusion and exclusion criteria. Third, the publication bias for each type of biomarker in this meta-analysis was minimal. Finally, we have included a wide range of popular but controversial biomarkers, ranging from biomedical trace elements to gut microbiota.

In conclusion, the present meta-analysis found an association between the levels of CRP, GABA, oxytocin, Fe, Zn and the relative abundance of *Bifidobacterium*, *Parabacteroides*, *Bacteroides*, *Clostridium,* and ASD, suggesting that these indications may be promising biomarkers for ASD. Future investigation is necessary to determine whether these biomarker profiles are specific to ASD. Additionally, our study also indicated that enhancing the levels of these biomarkers could serve as an effective intervention and treatment strategies for ASD.

## Data availability statement

The original contributions presented in the study are included in the article/Supplementary material, further inquiries can be directed to the corresponding author.

## Author contributions

PL: Conceptualization, Data curation, Writing – original draft, Supervision, Formal analysis, Investigation. QZ: Data curation, Formal analysis, Writing – original draft, Writing – review & editing. JuS: Formal analysis, Writing – original draft, Data curation, Investigation. QL: Data curation, Formal analysis, Writing – original draft, Investigation. DL: Data curation, Writing – review & editing. MZ: Data curation, Writing – review & editing. XF: Data curation, Writing – review & editing. LZ: Data curation, Writing – review & editing. MW: Data curation, Writing – review & editing. XL: Data curation, Writing – review & editing. QC: Data curation, Writing – review & editing. KL: Data curation, Writing – review & editing. YZ: Data curation, Writing – review & editing. CQ: Data curation, Writing – review & editing. ZL: Data curation, Writing – review & editing. PM: Data curation, Writing – review & editing. RW: Data curation, Writing – review & editing. HL: Supervision, Writing – review & editing. KD: Data curation, Writing – review & editing. XG: Supervision, Writing – original draft, Data curation, Investigation. XC: Conceptualization, Formal analysis, Supervision, Writing – original draft, Data curation, Investigation. YS: Conceptualization, Investigation, Methodology, Supervision, Writing – review & editing, Data curation. JiS: Formal analysis, Investigation, Methodology, Supervision, Validation, Writing – review & editing.
